# Modified C7 pedicle subtraction osteotomy for the correction of cervicothoracic kyphosis

**DOI:** 10.1186/s12891-020-3053-7

**Published:** 2020-01-14

**Authors:** Yichen Meng, Jun Ma, Lun Shu, Jia Yin, Rui Gao, Ce Wang, Xuhui Zhou

**Affiliations:** Department of Orthopedics, Changzheng Hospital, Second Military Medical University, 415 Fengyang Road, Shanghai, 200003 People’s Republic of China

**Keywords:** Pedicle subtraction osteotomy, Cervicothoracic kyphosis, Morphometric study, Case series, Ankylosing spondylitis

## Abstract

**Background:**

Osteotomies in the cervical spine are technically challenging. The purpose of this study was to evaluate the feasibility of the modified pedicle subtraction osteotomy (PSO) technique at C7 to be used for the treatment of cervicothoracic kyphosis secondary to ankylosing spondylitis.

**Methods:**

A total of 120 cervical spine computed tomography (CT) scans (of 82 male and 38 female patients) were evaluated. The scans were taken parallel to the middle sagittal plane and the sagittal plane intersecting the pedicles. Simulated osteotomy was performed by setting the apex of the wedge osteotomy at different points, and morphologic measurements were obtained. Seven patients with cervicothoracic kyphosis who underwent a modified PSO at C7 between May 2009 and June 2015 were retrospectively evaluated. The mean follow up was 32.9 months (range 21–54 months). Preoperative and postoperative chin-brow vertical angle (CBVA), sagittal vertical axis (SVA) and sagittal Cobb angle of the cervical region were reviewed. The outcomes were analyzed through various measures, which included the 36-Item Short Form Health Survey (SF-36) and a visual analog scale for neck pain.

**Results:**

In this morphometric study, a modified PSO was performed on 87 patients (59 male and 28 female) with a reasonable ratio of 72.5%. In the case series, radiographic parameters and health-related quality-of-life measures were found to show significant postoperative improvement in all patients. No major complications occurred, and no implant failures were noted until the latest follow up.

**Conclusions:**

The modified PSO is a safe and valid alternative to the classic PSO, allowing for excellent correction of cervical kyphosis and improvement in health-related quality-of-life measures.

## Background

Severe and rigid cervicothoracic kyphosis in patients with ankylosing spondylitis can cause pain, myelopathy, radiculopathy, marked limitation of horizontal gaze or upright posture, swallowing dysfunction leading to aspiration, as well as social impairment [1]. For these patients, surgical correction and osteotomies are often required. The osteotomies that can be performed in the cervical spine are largely similar to those performed in the thoracolumbar spine. However, osteotomies in the cervical spine are technically more challenging to perform, due to the need to protect the vertebral artery and the potentially devastating consequences of a cervical level cord injury. Several reports described the cervical pedicle subtraction osteotomy (PSO) technique and have demonstrated that it is more stable and safer than opening wedge osteotomy [2–6]. Cervical PSO includes the resection of the posterior column and a transpedicular wedge osteotomy, shortening the posterior and middle columns, without lengthening of the anterior column [7, 8]. Vertebral column decancellation (VCD) is a new thoracolumbar osteotomy technique, which was first described by Wang et al. in a series of nine patients with severe Pott’s kyphosis [9]. VCD was found to have several advantages over PSO. First, a PSO is performed using the retained anterior longitudinal ligament to act as a hinge to close the osteotomy [6]. This differs from the technique of a VCD, where the hinge of the correction is located at the border of the anterior and medial column [10]. If the correction is not adequate, a retroposition of the hinge can be performed [11]. Second, a PSO can achieve a mean correction of 30–40° according to the literature [12, 13], while VCD can achieve a mean correction of 83.8° [11]. In addition, VCD includes osteoclasis of the anterior cortex of the osteotomised vertebrae, which decreases the need for shortening of the posterior column, reducing the risk of neurological deficits [14]. However, this procedure has only been described for deformities of the thoracolumbar spine. Therefore, based on the technique of VCD, we modified PSO by shifting the hinge of correction backwards from the anterior longitudinal ligament.

The purpose of this study was, firstly, to explore the feasibility of the modified PSO technique for C7 by simulating osteotomy on computed tomography (CT) scanning, and secondly, to investigate the efficacy of the modified C7PSO technique on a series of patients with cervicothoracic kyphosis secondary to ankylosing spondylitis.

## Methods

A total of 120 cervical spine computed tomography (CT) scans (of 82 male and 38 female patients) were evaluated in this study. All patients had undergone standardized axial and sagittal bone-window CT scanning at C0-T3 in a supine neutral position. Patients with clinical and radiographic evidence of fracture, deformity, tumor or advanced degenerative changes were excluded.

For the CT examination, the same Siemens scanner (Siemens Healthcare, Erlangen, Germany) was used on all patients, with the following scan parameters: slice thickness, 0.6 mm; scan time, 52 s; 130 kV; and 120 mA/s.

**Fig. 1:**
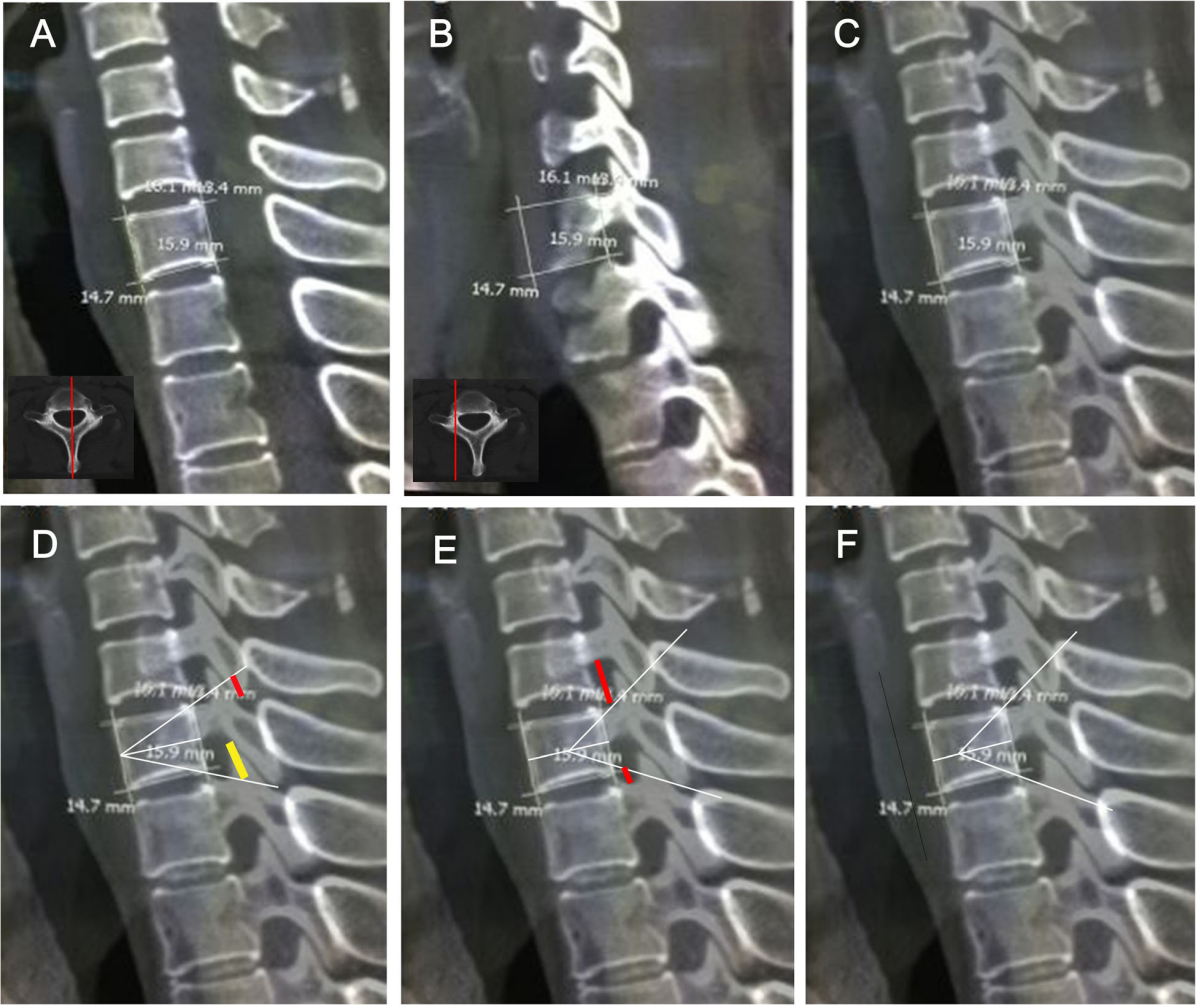
**a** CT scan at C7 showing measurements of vertebrae dimensions on the on the middle sagittal plane. **b** The outline of the vertebral body is projected parallel to itself to the sagittal plane intersecting the pedicles. **c** Merged A and B showing the pedicles and facets on the middle sagittal plane. **d**-**f** The apex of wedge osteotomy is set at different points to simulate different types of osteotomy: the intersection point of mid-vertebral body line with anterior vertebral body wall (**d**), the midpoint of mid-vertebral body line (**e**), the anterior 1/3 point of mid-vertebral line (**f**). CT indicates computed tomography

**Fig. 2:**
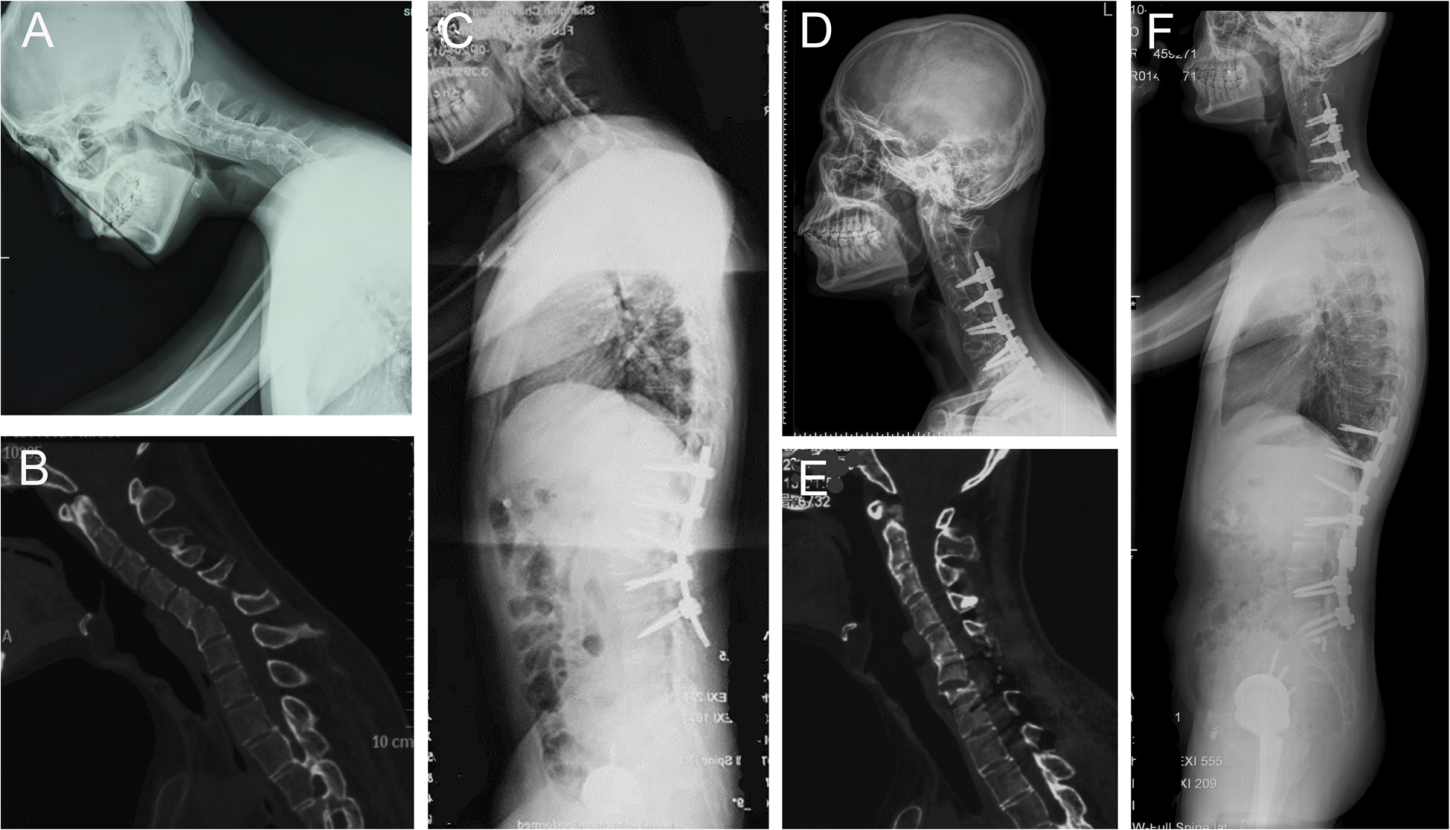
Pre- and post-operative radiological outcomes and clinical photographs. **a-c** A patient with cervicothoracic kyphosis secondary to ankylosing spondylitis presented with restriction of forward gaze. **d-f** Modified PSO was performed at C7, and radiographs at 2 years follow-up revealed significant improvement of the deformity

**Fig. 3:**
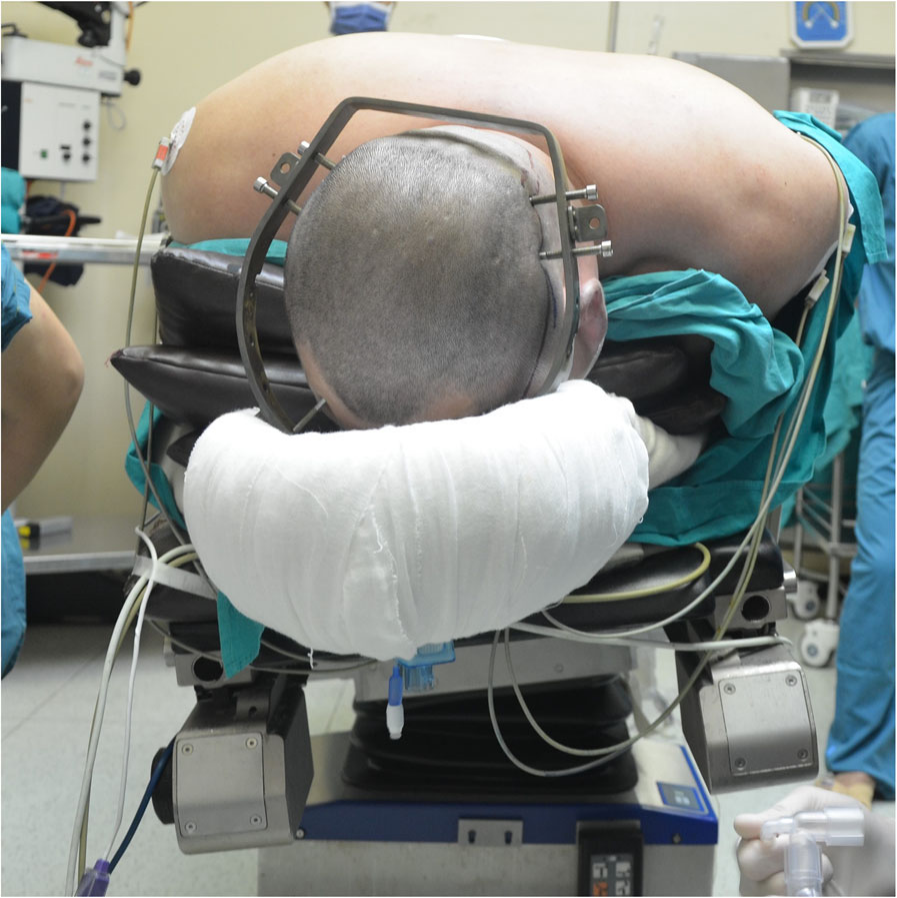
The patient is placed prone on a plaster bed on the operating table, with several rectangular paddings filled between his ventral side and the plaster bed

A quantitative evaluation of the morphology of the cervical spine regarding its feasibility for modified PSO was conducted. PSO was performed at C7, due to this location being a safe location for the vertebral artery in front of the transverse process of C7, the size of the spinal canal at C7-T1, the mobility of the spinal cord and eighth cervical nerves in this region and the probability of preservation of reasonable hand function if a C8 nerve root injury were to occur [15]. Simulated C7 osteotomy and measurements were made at 2 planes: the middle sagittal plane (Fig. 1a) and the sagittal plane intersecting the pedicles (Fig. 1b). On the middle sagittal plane, the outline of the C7 vertebral body was indicated using a quasi-rectangle, while parameters, including aVBH (anterior vertebral body height), pVBH (posterior vertebral body height), sVBD (superior vertebral body depth) and iVBD (inferior vertebral body depth) were assessed. Then, the apex of wedge osteotomy was set at different points to simulate different types of osteotomy: (1) the intersection point of the mid-vertebral body line with the anterior vertebral body wall (traditional PSO) (Fig. 1d); (2) the midpoint of the mid-vertebral body line (VCD) (Fig. 1e); (3) the anterior 1/3 point of the mid-vertebral line (modified PSO) (Fig. 1f). Osteotomy lines were straight lines that joined the apex and the posterior top and bottom corners of the quasi-rectangle. The angles formed between two osteotomy lines indicated the maximum correction of sagittal deformity that could be achieved by surgery. Then, the quasi-rectangle and osteotomy lines were projected parallel to itself onto the sagittal plane intersecting the pedicles (Fig. 1c-f). In order to investigate the optimal resected length of the articular process during the osteotomy, the length of the C6 inferior facet (red line in Fig. 1d) and T1 superior facet (yellow line in Fig. 1d) intercepted by the osteotomy lines were measured.

During the simulated osteotomy, two criteria needed to be fulfilled. First, the pedicles of C6 and T1 were important for cervical spine stabilization and should not be impinged by the simulated osteotomy line. Second, the C7 and C8 nerve roots should not be compressed on closure of the osteotomy. Kobayashi et al. reported that the mean values of the width at the entry of C7 and C8 in the vertebral foramen were 4.8 mm and 4.3 mm, respectively [16]. Therefore, after column closure following osteotomy, the distance from the lower C6 pedicle surface to the upper T1 pedicle surface in sagittal plane needed to be larger than 9.1 mm. The distance between the bone cortex of C6 pedicle inferior edge and the upper osteotomy line (longer red line in Fig. 1e) and the distance between the cortex of T1 pedicle superior edge and the lower osteotomy line (shorter red line in Fig. 1e) were measured. The sum of these two measurements is the theoretical distance between C6 and the T1 pedicle after wedge closure, and it is also the space within which the C7 and C8 nerve roots are accommodated after osteotomy.

All the above mentioned measurements were made manually by three independent authors (YM, JM and LS) using a digital cursor in CT scan software. Statistical analyses were performed using SPSS 19.0 software (SPSS Inc., Chicago, IL). Continuous variables are summarized as mean (± SD) or median (range).

In order to further confirm the feasibility of the modified PSO, 7 patients with cervicothoracic kyphosis secondary to AS, who had undergone a modified C7PSO at our institution, between May 2009 and June 2015, were retrospectively evaluated. A diagnosis of AS was made based on New York standards [17]. The 7 patients were aged between 26 and 45 years old. Indications for surgery included marked restriction of forward gaze, myelopathy and/or radiculopathy and progressive deformity. Six patients had a chin-on-chest deformity, while one patient suffered from persistent pain despite conservative treatment. Three patients had undergone previous surgery, including lumbar PSO and total hip replacement. Radiological outcomes were assessed using standing lateral radiographs, computed tomography (CT) scans with reconstructions and magnetic resonance imaging (MRI). Magnetic resonance angiography (MRA) was performed to confirm the position and flow of the vertebral artery. Clinical photographs were also obtained preoperatively and at final follow-up. The Chin Brow Vertical Angle (CBVA) was measured using these photographs [18]. Monitoring of somatosensory-evoked potentials and motor-evoked potentials was performed for all patients. This study was conducted in accordance with the tenets of the Declaration of Helsinki and was approved by the Institutional Review Board of Changzheng Hospital. Written informed consent was obtained from all participants prior to the study.

## Results

### Radiographic results of simulated osteotomy

The patients were aged between 21 and 46 years old, and included 82 male patients and 38 female patients. The parameters measured during simulated osteotomy are illustrated in Fig. 1 and Tables 1 and 2. In female patients, the mean ± SD sVBD and iVBD were 16.4 ± 1.4 mm and 16.0 ± 1.3 mm, respectively. In male patients, the mean ± SD sVBD and iVBD were 18.7 ± 1.3 mm and 18.3 ± 1.4 mm, respectively. There was a statistically significant difference in sVBD and iVBD based on gender (*P* < 0.05). In female patients, the mean ± SD aVBH and pVBH were 13.1 ± 1.0 mm and 13.4 ± 1.1 mm, respectively. In male patients, the mean ± SD aVBH and pVBH were 14.7 ± 1.5 mm and 14.8 ± 2.1 mm, respectively. There was a statistically significant difference in aVBH and pVBH based on gender (P < 0.05) (Table 1).

**Table 1:** C7 superior vertebral body depth, inferior vertebral body depth, anterior vertebral body height and posterior vertebral body height (in mm)

	N	sVBD	iVBD	aVBH	pVBH
Male	82	18.7 ± 1.3	18.3 ± 1.4	14.7 ± 1.5	14.8 ± 2.1
Female	38	16.4 ± 1.4	16.0 ± 1.3	13.1 ± 1.0	13.4 ± 1.1
Average		17.8 ± 1.7	17.4 ± 1.7	14.1 ± 1.5	14.2 ± 2.0

All values were presented as mean ± SD. *sVBD* Superior vertebral body depth, *iVBD* Inferior vertebral body depth, *aVBH* Anterior vertebral body height, *pVBH* Posterior vertebral body height

**Table 2:** Surgical parameters and feasibility of different kinds of simulated osteotomy

	tPSO			VCD			mPSO		
	Male	Female	All patients	Male	Female	All patients	Male	Female	All patients
Angle corrected (degree, mean ± SD)	43.4 ± 4.5	41.2 ± 3.7	41.9 ± 4.3	73.0 ± 7.1	69.4 ± 4.3	71.7 ± 5.7	60.1 ± 6.2	58.7 ± 5.4	59.1 ± 4.7
Resected length of C6 inferior facet (mm, mean ± SD)	3.3 ± 2.0	3.2 ± 1.7	3.2 ± 1.3	5.7 ± 2.3	5.1 ± 1.7	5.4 ± 2.0	4.7 ± 2.1	4.2 ± 1.1	4.5 ± 1.6
Resected length of T1 superior facet (mm, mean ± SD)	5.5 ± 2.5	5.3 ± 2.2	5.4 ± 2.2	7.5 ± 2.8	6.9 ± 2.5	7.2 ± 2.2	6.5 ± 2.6	6.2 ± 1.6	6.4 ± 2.1
Feasibility of simulated osteotomy									
No. of patients with C6/T1 pedicles impinged	3	4	7	33	18	51	10	9	19
No. of patients with C7/C8 nerve roots compressed	5	3	8	28	15	43	13	4	17
No. of simulated osteotomies considered to be successful	72	34	106(88.3%)	26	12	38(31.7%)	59	28	87(72.5%)

*tPSO* Traditional pedicle subtraction osteotomy, *VCD* Vertebral column decancellation, *mPSO* Modified pedicle subtraction osteotomy

When the apex of wedge osteotomy was set at the intersection point of the mid-vertebral body line with the anterior vertebral body wall (traditional PSO) (Fig. 1d), the simulated osteotomy could achieve a mean ± SD correction angle of 41.2 ± 3.7° in females and 43.4 ± 4.5° in males. In female patients, the resected lengths of the C6 inferior facet and the T1 superior facet were 3.2 ± 1.7 mm and 5.3 ± 2.2 mm, respectively. In male patients, the resected lengths of the C6 inferior facet and the T1 superior facet were 3.3 ± 2.0 mm and 5.5 ± 2.5 mm, respectively. In total, 106 patients (72 male and 34 female) who met the criteria of neither C7/C8 nerve roots nor the C6/T1 pedicles being impinged were considered to have undergone a successful osteotomy.

When the apex of wedge osteotomy was set at the midpoint of mid-vertebral body line (VCD) (Fig. 1e), the simulated osteotomy achieved a mean ± SD correction angle of 69.4 ± 4.3° in females and 73.0 ± 7.1° in males. In female patients, the resected lengths of the C6 inferior facet and the T1 superior facet were 5.1 ± 1.7 mm and 6.9 ± 2.5 mm, respectively. In male patients, the resected lengths of the C6 inferior facet and the T1 superior facet were 5.7 ± 2.3 mm and 7.5 ± 2.8 mm, respectively. This osteotomy technique was found to be feasible for 38 patients (26 male and 12 female).

When the apex of wedge osteotomy was set at the anterior 1/3 point of mid-vertebral line (modified PSO) (Fig. 1f), the simulated osteotomy could achieve a mean ± SD correction angle of 58.7 ± 5.4° in females and 60.1 ± 6.2° in males. In female patients, the resected lengths of the C6 inferior facet and the T1 superior facet were 4.2 ± 1.1 mm and 6.2 ± 1.6 mm, respectively. In male patients, the resected lengths of the C6 inferior facet and the T1 superior facet were 4.7 ± 2.1 mm and 6.5 ± 2.6 mm, respectively. Nineteen patients had their C6/T1 pedicles touched by the osteotomy lines and 17 patients had inadequate room for C7 and C8 nerve roots after closure of the osteotomy. When combined together, the results found that a total of 87 patients (59 male and 28 female) had undergone a successful simulated osteotomy.

### Clinical results of case series

Osteotomy was performed at C7 on all patients. Demographic and follow up data are summarized in Tables 3 and 4. The mean follow up period was 32.9 months (range, 21–54 months). The average operation time was 255 min (range, 220–310 min). The mean estimated blood loss per patient was 1475 ml (range, 1200–2000 ml). C2-T1 kyphosis improved from a preoperative mean of 26.3° (range, 16–34°) to a postoperative mean of − 5.4° (range, − 17-5°), which is statistically significant (*P* < 0.05). The mean CBVA improved from 43.7° (range, 35–50°) to − 0.9° (range, − 7-15°). Preoperatively, the mean C2-T1 sagittal imbalance was preoperatively 6.6 cm (range, 5.5–8 cm), which was postoperatively significantly corrected to 3.0 cm (range, 2.2–3.8 cm), with a mean regional correction of 3.7 cm (45.1%). A statistically significant decrease was found in the VAS scores (8.6 to 1.7, P < 0.05). The mean physical component summary scores of SF-36 improved from a preoperative score of 20.7 to a score of 53.3 at final follow-up, which is statistically significant (P < 0.05). No permanent neurological deficit was reported in any patient. One patient reported of transient postoperative deterioration of neurological function after surgery, but his symptoms were relieved after 2 weeks of conservative treatment. One patient suffered a deep infection, which was successfully treated using debridement and sensitive intravenous antibiotics. No complications related with the placement of hardware were reported.

**Table 3:** Demographic and operative data

Case	Gender	Follow-up (months)	Diagnosis	Previous surgery	Pedicle screw placement	Operation time (min)	Blood loss (ml)	Complications
1	M	33	CTJ kyphosis w/ chin-on-chest deformity	–	C4–6, T1–3	260	1200	–
2	M	54	CTJ kyphosis w/ chin-on-chest deformity	Lumbar PSO and total hip replacements	C4–6, T1–3	230	1400	–
3	M	23	CTJ kyphosis w/ chin-on-chest deformity	Lumbar PSO	C4–6, T1–3	230	1350	–
4	M	26	CTJ kyphosis and cervical spondylosis	–	C4–6, T1–3	220	1670	–
5	M	21	CTJ kyphosis w/ chin-on-chest deformity	–	C4–6, T1–3	280	1300	Transient neurological deficit
6	M	40	CTJ kyphosis w/ chin-on-chest deformity	Total hip replacements	C4–6, T1–3	260	1410	Deep infection
7	M	33	CTJ kyphosis w/ chin-on-chest deformity	–	C4–6, T1–3	310	2000	–

**Table 4:** Correction of deformity and follow-up data

Case	C2-T1 kyphosis (°)			CBVA (°)			C2-T1 SVA (cm)			VAS		PCS score	
	Preop	Postop	Correction	Preop	Postop	Correction	Preop	Postop	Correction	Preop	Follow-up	Preop	Follow-up
1	34	5	29	50	15	35	7	2.5	4.5	9	3	21	52
2	24	−14	38	48	−3	51	8	3.8	4.2	9	1	23	53
3	29	3	26	43	−2	45	7	3.4	3.6	8	0	20	52
4	27	3	24	40	−3	43	6	2.9	3.1	8	2	17	54
5	28	−7	35	35	−1	36	7	3.3	3.7	8	2	22	53
6	16	−17	33	44	−5	49	5.5	2.2	3.3	9	3	21	54
7	26	−11	37	46	−7	53	6	2.7	3.3	9	1	21	55
Mean	26.3	−5.4	31.7	43.7	−0.9	44.6	6.6	3.0	3.7	8.6	1.7	20.7	53.3

*CBVA* Chin brow vertical angle, *SVA* Sagittal vertical axis, *VAS* Visual Analog Scale, *PCS* Physical component summary

## Discussion

Cervical osteotomy performed for ankylosing spondylitis remains challenging due to the high risk of neurovascular compromise. In 1958, Urist first reported on a case of cervical kyphosis treated using an opening wedge osteotomy similar to the Smith-Petersen osteotomy [15]. This technique was able to achieve significant correction of deformity but involved lengthening of the anterior column, which potentially threatened anterior soft tissue structures, such as trachea and esophagus). In 2007, Tokala et al. first described the cervical PSO procedure [6]. Similar to PSO on the thoracolumbar spine, PSO on the cervical spine has the advantages of structural stability and a wider cancellous contact surface for bony union, over extension-type osteotomy [19]. However, in some cases, a single-stage posterior PSO may not be suitable to correct extremely severe kyphotic deformity. VCD is a relatively new technique for spinal osteotomy, which was first described in a series of nine patients with severe thoracolumbar kyphosis [9]. Nevertheless, the use of this technique on the cervical spine has not been described. In this study, we modified the cervical PSO by shifting the hinge of correction backwards to the anterior 1/3 of the C7 vertebral body. Measurements of vertebrae dimensions provided by CT scanning determined the operating space for the osteotomy. We defined the anatomic morphometry of the C7 vertebra based on gender. The VBD and VBH determined the antero-posterior and supero-inferior diameter, respectively, for each vertebral body. The results of our study show that the average C7 VBD and VBH values are similar to those obtained by Keskin [20], Koller [21] and Chen [22]. We also found significant differences between genders for these parameters.

Instead of a “V” shaped osteotomy, the mPSO was a “Y” shaped osteotomy. In theory, the mPSO could achieve greater correction using the same resection in height of the posterior wall. Based on the results of the simulated osteotomy, mPSO was determined to be able to obtain a mean of 59.1° of correction at the osteotomized level, while PSO provided a correction of 41.9°. However, in our series, the results show that we achieved a mean correction of 31.6° by performing mPSO. This finding is inconsistent with the average of 40–60° correction achievable using classic PSO [3, 6]. Possible explanations are that both Tokala and Deviren manually extended the head and closed the wedge osteotomy using a Mayfield clamp or halo ring. In our practice, we performed in situ rod bending to restore cervical lordosis, which yielded a limited result. Second, the cases reported on in our research (average age 36.8 years old) were much younger than those reported on by Deviren (average age 70 years old). In general, younger patients have less cervical lordosis and Deviren et al. needed a larger degree of osteotomy to maintain normal cervical alignment. Third, most of the patients had a loss of horizontal gaze as the chief complaint. Thus, restoration of the normal visual field has become the main purpose of cervical osteotomy. We used CBVA measurements as the basis for kyphosis correction and evaluation of treatment outcome. Therefore, we individualized the kyphosis correction plan based on the CBVA of each patient. For the illustrative case, the simulated correction angle was larger than the actual correction angle required when we set the apex of wedge osteotomy at the anterior 1/3 point of the mid-vertebral line. As a result, we finally set the apex of wedge osteotomy at approximately the anterior 1/5 point during the surgery and achieved a satisfactory outcome (CBVA decreased from 48° to − 3°).

Mehdian et al. implied of the importance of assessing the contribution of the hips, thoracolumbar spine, and the cervicothoracic spine to overall flexion deformity. They also suggested that a severely compromised frontal visual field from cervical kyphosis was best addressed by performing a cervical osteotomy [23]. The Illustrative case shown in Fig. 2 presented with sagittal imbalance, restricted frontal visual field and severe flexion deformities of the hips. Total hip replacement was performed based on his complaint. Next, a lumbar osteotomy was performed and improved forward vision to some extent but was not sufficient to restore horizontal gaze. Finally, a cervical osteotomy was performed, and the patient was able to maintain his frontal visual field. Simmons et al. recommended that slight under correction of kyphosis deformity was more beneficial to patients than overcorrection, since there needs to be a compromise between being able to look straight ahead when standing and walking, and still being able to work at a desk or drive a car [24]. In their practice, they usually corrected flexion deformity to approximately 10° of the flexion. We achieved dramatic CBVA correction with a mean angle of 44°.

The size of C6 and T1 facets to be excised should be carefully assessed before surgery and intraoperatively to avoid any compression of the nerve roots. Increased resection area provides ample space for osteotomy and instrumentation but may result in inadequate contact of the posterior column on closure of the osteotomy site, causing a gap, which may have an impact on the stability of the posterior column. In our morphometric measurements, the resected lengths of the C6 inferior facet and the T1 superior facet were 4.7 ± 2.1 mm and 6.5 ± 2.6 mm, respectively, for males. Intraoperatively, the C6 inferior facet or the T1 superior facet should be further removed, if the C7 and C8 nerve roots are not free. Reduction of the deformity was found to be the most critical step for a successful osteotomy, while intraoperative manual reduction was found to be the most basic and the most commonly reported technique [6, 23–25]. This technique required that the surgeon hold a Mayfield clamp or halo ring, while an assistant monitored the spinal dura and nerve roots. In our study, a plaster bed was placed on the Jackson table and the patient was placed in the prone position on the plaster bed with several rectangular paddings filled between his ventral side and the plaster bed (Fig. 3). After osteotomy, with the head fixed, the assistant closed the osteotomy site by manually holding the shoulders of the patient and extending the neck, while another assistant removed the uppermost padding. This procedure was repeated multiple times to obtain the desired correction angle.

This study was limited by a small sample size of cases, since severe cervicothoracic kyphosis following ankylosing spondylitis is a rare condition. However, previous studies have reported on only a few cases undergoing cervical PSO [3, 6], and our study reported on the largest number of cases with cervicothoracic kyphosis following ankylosing spondylitis.

## Conclusions

In this study, we concluded that mPSO for C7 was feasible based on CT morphometric measurements. The series confirmed that mPSO was a valid alternative to classic PSO, allowing easier achievement of the correction goals.

## Data Availability

The data supporting the findings of this study are available from the last author (XZ, zhouxuhui@smmu.edu.cn) upon reasonable request.

## Abbreviations

aVBH: Anterior vertebral body height
CBVA: Chin-brow vertical angle
CT: Computed tomography
iVBD: Inferior vertebral body depth
PSO: Pedicle subtraction osteotomy
pVBH: Posterior vertebral body height
SF-36: 36-Item Short Form Health Survey
SVA: Sagittal vertical axis
sVBD: Superior vertebral body depth
VAS: Visual analog scale
VCD: Vertebral column decancellation
